# Tolterodine is a novel candidate for assessing CYP3A4 activity through metabolic volatiles to predict drug responses

**DOI:** 10.1038/s41598-025-86450-9

**Published:** 2025-01-20

**Authors:** Valentina Stock, Rebecca Hofer, Franziska Lochmann, Vera Spanke, Klaus R. Liedl, Jakob Troppmair, Thierry Langer, Hubert Gstach, Christian Dank, Chris A. Mayhew, Sarah Kammerer, Veronika Ruzsanyi

**Affiliations:** 1 Institute for Breath Research, University of Innsbruck, Innrain 80/82, Innsbruck, 6020 Austria; 2 Institute for Theoretical Chemistry, University of Innsbruck, Innrain 80/82, Innsbruck, 6020 Austria; 3 Daniel Swarovski Research Laboratory, Department of Visceral, Transplant and Thoracic Surgery, Medical University of Innsbruck, Innrain 66, Innsbruck, 6020 Austria; 4 Department of Pharmaceutical Chemistry, University of Vienna, Althanstraße 14, Vienna, 1090 Austria; 5 Institute of Organic Chemistry, University of Vienna, Währinger Straße 38, Vienna, 1090 Austria; 6 Institute of Biotechnology, Molecular Cell Biology, Brandenburg University of Technology Cottbus- Senftenberg, 01968 Senftenberg, Germany

**Keywords:** Breath test, CYP3A4, HepG2, Tolterodine, Acetone, Volatile biomarkers, Enzymes, Enzyme mechanisms, Enzymes, Metabolic pathways, Metabolomics, Small molecules, Bioanalytical chemistry, Mass spectrometry

## Abstract

Cytochrome P450 (CYP) 3A4 plays a major role in drug metabolism. Its activity could be determined by non-invasive and cost-effective assays, such as breath analysis, for the personalised monitoring of drug response. For the first time, we identify an isotopically unlabelled CYP3A4 substrate, tolterodine that leads to the formation of a non-toxic volatile metabolite, acetone, which could potentially be applied to monitor CYP3A4 activity in humans. In vitro biotransformation of tolterodine by HepG2 cells overexpressing CYP3A4, CYP2D6 or CYP2C9 was investigated by LC-MS analysis of cell culture supernatant for the non-volatile metabolite, N-dealkylated tolterodine, and PTR-ToF-MS analysis of the headspace for acetone. The highest level of the N-dealkylated metabolite was produced by HepG2-CYP3A4. Concentration dependent effects of tolterodine were analysed, resulting in TC_50_ values of 414 µM and 375 µM for HepG2-CYP3A4 and reference cells, respectively. Acetone and N-dealkylated tolterodine levels increased continuously over 24 h in HepG2-CYP3A4. Treatment with either a pan-CYP inhibitor, 1-aminobenzotriazole, or a CYP3A4 inhibitor, ketoconazole, considerably reduced the production of both metabolites in HepG2-CYP3A4 cells. These findings pave the way for the further development of non-invasive breath tests using unlabelled precursors to determine CYP enzyme activity in individuals.

**Supplementary Information:**

The online version contains supplementary material available at 10.1038/s41598-025-86450-9.

## Introduction

Drug metabolism entails the body’s ability to metabolise and break down pharmaceuticals, a pivotal factor in determining drug efficacy and safety. However, owing to factors such as cost and process complexity, determining or monitoring individual drug metabolism is typically reserved for exceedingly uncommon cases.

The design and dose needed for the majority of drugs are based on the average responses observed in clinical trials. Although this approach is sufficient for some patients, personalised monitoring may be required for a significant number of individuals due to their distinct metabolic profiles resulting from genetic variances, existing health conditions, or drug-drug interactions. Factors such as age, diet, drinking, smoking and other life-style behaviour can also significantly impact drug metabolism, thereby influencing a drug’s efficacy or elevating the risk of adverse effects^1^. Consequently, there is a need for fast, non-invasive and cost-effective tests for predicting drug metabolism in order to customise treatments according to individual requirements that provides an alternative to the existing strictly genotyping-based approaches for cytochrome P450 (CYP) enzymes^2^.

The analysis of metabolic volatiles in breath is considered a promising tool for non-invasive diagnostics. Volatile metabolites in exhaled air have the potential to provide a comprehensive picture of whole-body metabolism. However, only a few breath tests are currently in clinical use. These tests use specific ingested substrates to target selected metabolic pathways. The most established of these tests is the one which assesses carbohydrate malabsorption. In this test, the patient is required to ingest the respective sugar, after which the change in hydrogen concentrations in the breath is monitored^3,4^. Another commonly used clinical breath test involves the use of ^13^C-urea to diagnose *Helicobacter pylori *infections in the stomach^5^. This is currently the only ^13^C-labelled breath test that has been approved by the FDA^6^. Several other breath tests have been developed using ^13^C-labelled precursors with the aim of predicting individual therapeutic responses by targeting various CYP enzymes^7,8^, such as the ^13^C-erythromycin breath test for CYP3A^9^. These CYP enzymes are responsible for the metabolism of the majority of pharmaceuticals^10–13^.

Among the major CYP isoforms, CYP3A4 plays a major role, because it counts for up to 50% of the total drug metabolism and accounts for 30% of the relative abundance of hepatic CYPs^14–16^. It exhibits a broad substrate specificity and is responsible for the oxidation of many therapeutic drugs and a variety of structurally unrelated compounds, including steroids, fatty acids and xenobiotics. The variability of substrate metabolism between individuals probably occurs due to the inducibility of the CYP3A4 gene by drugs and environmental chemicals^17^. A considerable number of pharmaceuticals, including chemotherapeutics, antidepressants, antipsychotics, opiates, Ca^2+^channel blockers, protease inhibitors and sex hormones, are substrates of CYP3A4^18^. Therefore, there is considerable interest in targeting CYP3A4 for the development of assays that can predict individual drug and therapy responses. Whilst monitoring the expression of a number of CYP genes for routine purposes has become available^19^, the exact phenotype that is influenced by personal factors of an individual cannot be deduced exclusively from the genotype information.

Focusing on non-invasive phenotypic assays by means of breath analysis, there are several essential requirements for a selected substrate that have to be considered prior to successful application. The most important ones are that (i) isotopic labelling is not required (to reduce costs), (ii) its uptake by the organism is efficient, (iii) it is non-toxic, (iv) it has an increased selectivity towards the CYP of interest, and (v) its metabolism leads to a volatile metabolite^20,21^.

**Fig. 1:**
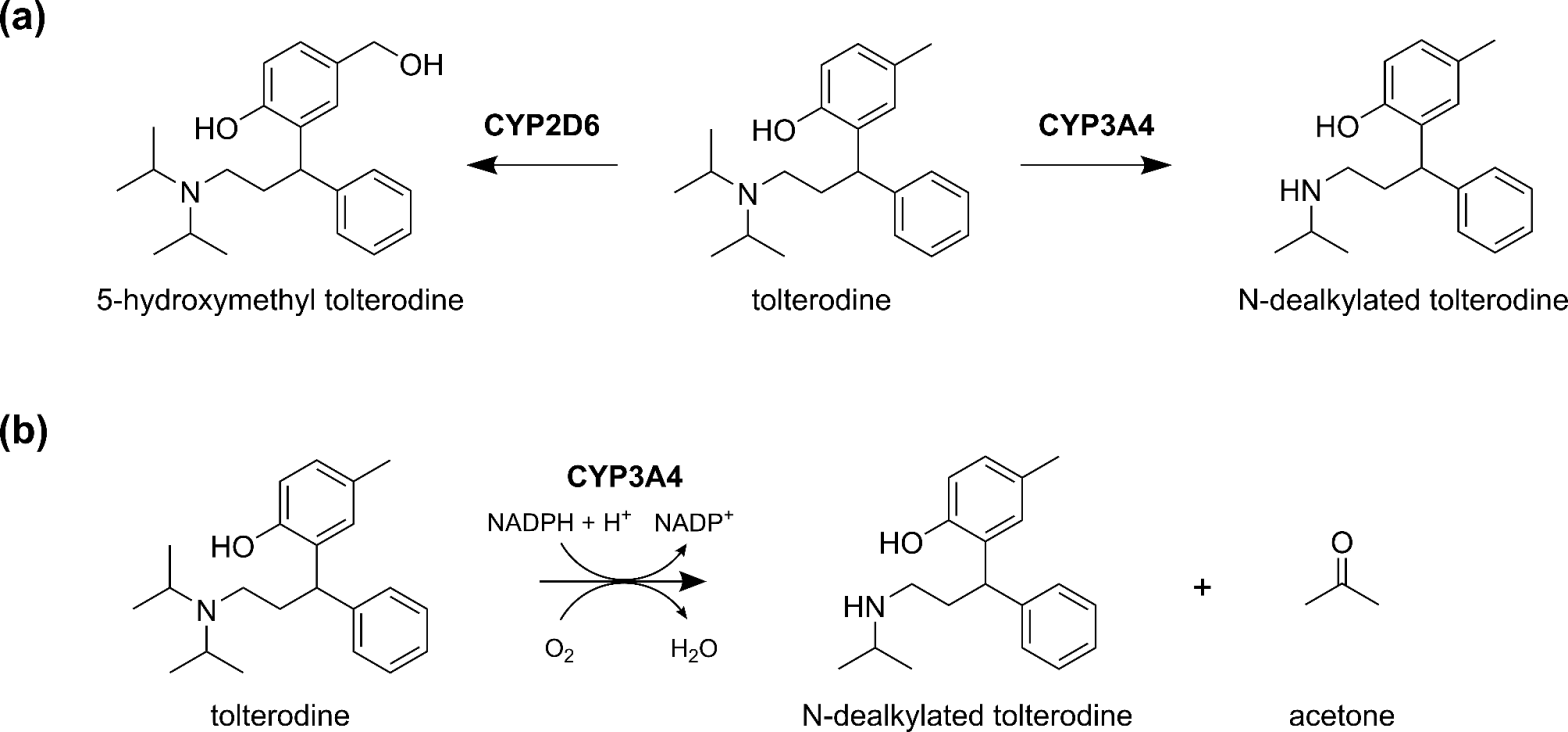
Illustrating (**a**) the main metabolic pathways for tolterodine^28^ and (**b**) the metabolism (N-dealkylation) of tolterodine by CYP3A4 enzymes that leads to our proposed production of acetone.

The liver is the predominant site of CYP expression, playing a key role in the clearance of xenobiotic compounds and in the control of systemic drug levels. The main chemical processes involved are oxidation, reduction and hydroxylation as phase I reactions^22–24^, resulting in an increase in the polarity (that might be further increased by phase II reactions) of the metabolite to become more hydrophilic for an easier clearance from the body via urine or stools^10,25,26^. In order to gain volatile metabolites during the catalysis by CYP3A4, the N- or O-dealkylation reactions should be the focus of interest. We therefore selected the substrate tolterodine (C_22_H_31_NO), which is a medication commonly used to treat symptoms of an overactive bladder^27^, that is primarily metabolised by CYP2D6 and CYP3A4 through hydroxylation / dealkylation to 5-hydroxymethyl tolterodine or 4-methyl-2-[1-phenyl-3-(propan-2-ylamino)propyl]phenol (N-dealkylated tolterodine) (Fig. 1a)^28^. Importantly, the N-dealkylation route leads to the N-dealkylated tolterodine (Fig. 1b) which means that acetone must be produced as a volatile metabolite through the cleavage of the isopropyl group from the nitrogen^28,29^.

Convenient methods for obtaining information about the biotransformation of a substrate, the enzymes involved and the production of new compounds divorced from the complexity of the human body are in vitro studies using mainly cultured human hepatocytes^30^. Human hepatoblastoma (HepG2) cells exhibit a low expression of the majority of CYP enzymes^11,31,32^. However, genetic modification of these cells allows enhanced expression of CYP isoforms, such as CYP3A4, and enables metabolic experiments focused on a particular isoform.

The objective of our research programme is to find suitable substrates that once metabolised produce volatiles that will appear in exhaled breath. Information obtained would help in the development of non-invasive metabolic tests that align with the requirements mentioned above. A major goal of this study is to identify for the first time whether acetone results from the metabolism of tolterodine by CYP3A4 as proposed in our metabolic pathway (Fig. 1b). This study therefore significantly moves forward the in vitro workflow developed by us in an earlier investigation that did not involve the production of volatile metabolites^21^. We have specifically investigated the specificity of the substrate tolterodine for CYP3A4, CYP2D6 and CYP2C9 using HepG2 clones overexpressing the respective isoforms. Further, the effects of time and tolterodine concentrations on the levels of the metabolites in the supernatant and headspace of the cell culture were determined, and the influence of 1-aminobenzotriazole and ketoconazole as CYP inhibitors on tolterodine biotransformation was investigated.

## Materials and methods

### Reagents and chemicals

Tolterodine L-tartrate (> 98.0%), acetone (> 99.5%), ketoconazole (> 98.0%) and 1-aminobenzotriazole (1-ABT, > 98.0%) were purchased from Tokyo Chemical Industry (TCI chemicals, Tokyo, Japan). The non-volatile metabolite N-dealkylated tolterodine (tolterodine impurity E CRS) was obtained from LGC standards (Wesel, Germany). The metabolite 5-hydroxymethyl tolterodine (99.05%) was supplied by MedChemExpress (Monmouth Junction, NJ, USA). Methanol (HPLC grade, ≥ 99.9%), dimethyl sulfoxide (DMSO, 99.9%), Krebs-Henseleit-Buffer modified (KHB), trypan blue solution (0.4%), 4-(2-hydroxyethyl)piperazine-1-ethanesulfonic acid (HEPES, > 99.5%) and calcium chloride dihydrate were purchased from Sigma Aldrich (St. Louis, MO, USA). Acetonitrile (ACN) (LiChrosolv®, ≥ 99.9%) was supplied together with sodium hydrogen carbonate (> 99.5%) from Merck (Darmstadt, Germany). Dulbecco’s modified Eagle’s medium (DMEM) was obtained from Pan Biotech (Aidenbach, Germany). Fetal bovine serum (FBS), L-glutamine and TrypLE reagent were purchased from Gibco BRL (Paisley, UK). Phosphate buffered saline (PBS) was obtained from Lonza (Basel, Switzerland). Blasticidin was supplied by PAA Laboratories GmbH (GE Healthcare, Chicago, IL, USA) and zeocin was purchased from Thermo Fisher Scientific (Waltham, MA, USA).

Water was obtained from a Millipore Milli-Q Biocel water purification system (Merck-Millipore, Billerica, MA, USA). KHB (pH 7.4) supplemented with 25 mM sodium hydrogen carbonate, 2 mM calcium chloride dihydrate and 25 mM HEPES was freshly prepared every 1–2 months and stored at 4 °C.

### Substrate selection

We conducted a systematic search of DrugBank^33^ to identify potential substrates for testing. DrugBank contains entries for approved or previously approved drugs, all of which have undergone extensive evaluation for adverse effects and ADME (absorption, distribution, metabolism, and excretion) criteria.

In the initial step, we collected all drugs listed as substrates of CYP3A4 and excluded those reported to induce or inhibit CYP3A4. For each remaining substrate, we retrieved the Simplified Molecular Input Line Entry System (SMILES) notation^34,35^. To refine our search, we focused on substrates containing an isopropylamine substructure, as the nitrogen-carbon bond in this group represents a potential site for CYP3A4-mediated oxidation, potentially yielding a ketone and an amine as metabolites.

Our analysis prioritized substrates that produced ketone metabolites due to practical challenges in detecting aldehydes and their subsequent oxidation products (acids). We translated the isopropylamine substructure into SMILES Arbitrary Target Specification (SMARTS) format^36^ and used RDKit (version 2019.03.2) to convert the SMARTS query into an RDKit substructure. We then screened the previously collected SMILES notations to identify matches with the isopropylamine substructure. Substrates containing this substructure were saved for further analysis.

The resulting list of substrates was manually curated through visual inspection. During this process, we ensured that the predicted ketone metabolite would completely disconnect from the remainder of the substrate upon metabolism. To simplify analysis, we further limited the ketone metabolites to those with no more than eight carbon atoms. Finally, we excluded substrates whose ketone metabolites contained hydrophilic groups, such as hydroxyl or carboxyl groups. For the identified substrates, we re-evaluated whether the desired metabolites were indeed produced through CYP3A4-mediated metabolism.

### Cell culture

HepG2 cell clones overexpressing CYP3A4, CYP2D6 and CYP2C9 as well as their corresponding empty vector (EV) control cell lines were provided by the Institute of Biotechnology, Molecular Cell Biology Group, Brandenburg University of Technology Cottbus-Senftenberg, Senftenberg, Germany^37,38^. All cell lines were cultivated in DMEM containing 2 mM L-glutamine and 10% FBS at 37 °C and 5% CO_2_ in a humidified incubator (CB 170, Binder, Tuttlingen, Germany). Upon reaching approximately 80% confluence, cells were passaged every five to six days.

In order to maintain high level expression in our HepG2 clones, the medium was supplemented every 3–4 weeks (for 2–3 days) with 3 µg mL^−1^ of blasticidin for the CYP3A4 overexpressing cells and the corresponding EV cells and 300 µg mL^−1^ of zeocin for the CYP2D6 and CYP2C9 overexpressing cells as well as for their corresponding EV cells. Cells were regularly examined for mycoplasma infections. Concentrations of DMSO, which was used as a solvent for various solids, did not exceed 1% in the procedures.

#### Determining tolterodine conversion in different HepG2 cell clones

 HepG2-CYP3A4, -CYP2D6 and -CYP2C9 overexpressing cells as well as their corresponding EV cells (5 × 10^5^ per well) were seeded into 24-well plates 24 h before treatment. Following medium change and washing with KHB, cells were exposed to 100 µM tolterodine diluted in KHB for 4 h at 37 °C and 5% CO_2_. After incubation, the cell supernatant was collected for liquid chromatography-mass spectrometry (LC-MS) analysis as described in “Sample preparation”.

#### Concentration-dependent effects of tolterodine

HepG2-CYP3A4 overexpressing cells and corresponding EV cells (5 × 10^5^ per well) were seeded into 24-well plates 24 h before treatment. Following medium change and washing with KHB, the cells were exposed to different concentrations (0–500 µM) of tolterodine diluted in KHB for 4 h at 37 °C and 5% CO_2_. After incubation, the cell supernatant was collected as described in “Sample preparation”. Cells were then washed with KHB and collected by trypsinisation. To investigate the possible toxic effects of tolterodine on cells, the trypan blue exclusion assay was applied. Aliquots of cell suspensions were mixed with an equal volume of trypan blue and counted using a Neubauer hemocytometer (Neubauer, Wien, Austria) in order to determine the fraction of viable cells.

#### Bioconversion of tolterodine to acetone

HepG2-CYP3A4 overexpressing cells and corresponding EV cells were seeded into 24-well plates (5 × 10^5^ per well; for analysis of the non-volatile metabolite) 24 h before treatment or in T75 flasks (2 × 10^6^ per flask; for analysis of the volatile metabolite, see below) four days before treatment to reach a confluency of 90% and thus, a high sample volume.

Following medium change and washing with KHB, the cells were exposed to 100 µM tolterodine diluted in KHB at 37 °C and 5% CO_2_. Incubation time differed according to the experimental setup: to investigate the time dependence of tolterodine metabolite production, incubation times ranged from 0 to 24 h; to investigate CYP inhibitors as described below, the incubation time was set at 4 h.

Two different CYP enzyme inhibitors were tested: (i) 1-ABT, a non-specific cytochrome P450 inhibitor^39^, and (ii) ketoconazole, a selective CYP3A4/5 inhibitor^40^. HepG2-CYP3A4 overexpressing cells and corresponding EV cells were seeded as described above and 100 µM tolterodine in KHB were added together with either 1000 µM 1-ABT or 5 µM ketoconazole (ketoconazole alone was pre-incubated for 10 min at 37 °C).

**Fig. 2:**
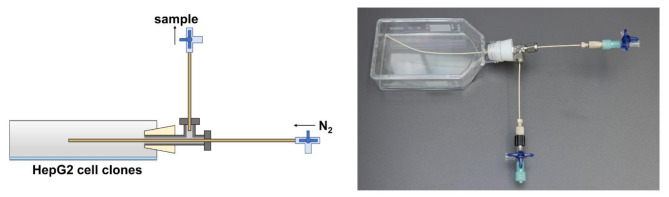
Design of the set-up used for headspace sampling.

To measure the production of the volatile metabolite, acetone, the cell culture flask had to be gas tight to prevent any acetone loss. Therefore, a gas tight device for the sampling of the headspace above the cells was designed. For this purpose, a silicone stopper (26.5 mm upper diameter, 21 mm lower diameter, 27 mm height) was inserted into the cell culture flask and a stainless-steel T-piece (Swagelok, 1/8″, Solon, OH, USA) was attached to the silicone stopper. A polyether ether ketone (PEEK) capillary (1/16″ OD and 0.75 mm ID) was inserted through the T-piece with one end placed in the headspace volume of the cell culture flask. The other end of the PEEK capillary was closed by a three-way-valve (Discofix® C, B.Braun, Melsungen, Germany), which served as a nitrogen gas inlet for headspace sampling. Another PEEK capillary with a three-way-valve was attached to the remaining side of the T-piece connector for the headspace sample outlet as illustrated in Fig. 2. To ensure that cells were not affected by the sealing of the flask, cell viability was analysed after respective incubation times using the trypan blue assay in sealed flasks and compared to cells in flasks treated the same way but without sealing. Cell viability did not differ between the two conditions.

After incubation, the cell headspace was collected for Proton Transfer Reaction-Time of Flight-Mass Spectrometer (PTR-ToF-MS) analysis as described in “Sample preparation” and the cell supernatant was investigated as explained in “Sample preparation”.

#### Equilibrium between gas and liquid phase

To investigate the levels of acetone in the buffer solution to that in the headspace, an acetone solution was prepared corresponding to the amount of N-dealkylated metabolite formed during bioconversion after 4 h and 6 h. Accordingly, 2.2 µM and 3.9 µM of acetone in KHB were added to a cell culture flask and sealed with the sampling device. Incubation was performed for 2 h in the cell incubator at 37 °C and 5% CO_2_ to reach an equilibrium between the gas and aqueous phases. Following incubation, the sample was measured as described in “Sample preparation”.

### Quantification of non-volatile tolterodine metabolites

#### Preparation of standard solutions

To consider matrix effects and ion suppression for LC-MS measurements by the KHB, calibration samples were also prepared in KHB and treated under the same conditions as the cells. Calibration standards of tolterodine (between 0.5 µM – 12 µM) and N-dealkylated tolterodine (between 0.005 µM − 5 µM) were freshly prepared by serial dilution of the corresponding 10 mM stock solution in KHB/ACN in a dilution ratio of 1:1. Each concentration was measured by LC-MS.

#### Sample preparation

After a given incubation time, the cell supernatant was collected, the reaction was stopped by addition of ice-cold ACN (1:1 ratio) and samples were centrifuged (Eppendorf 5415 R microcentrifuge, Eppendorf, Hamburg, Germany) at 16,100 g (relative centrifugal field) for 5 min. The mixture was then filtered through a syringe filter (PTFE, 0.2 μm pore size, Agilent Technologies, Santa Clara, CA, USA) to remove any solids. A part of the sample was then diluted tenfold. These diluted and undiluted samples were measured by LC-MS.

#### LC-MS analysis

Analytical measurements of tolterodine, N-dealkylated tolterodine and 5-hydroxymethyl tolterodine were performed on a Vanquish HPLC Flex-system with a binary pump coupled to an Orbitrap Q-Exactive mass spectrometer (both from Thermo Fisher Scientific). The samples were separated on a ZORBAX Eclipse XDB-C18 column (1.8 μm, 2.1 × 100 mm, Agilent Technologies) protected by a ZORBAX Eclipse Plus C18 guard column (1.8 μm, 2.1 × 5 mm, Agilent Technologies) at 40 °C. The mobile phase used for separation was a composition of formic acid 0.1% (v/v) in water (eluent A) and formic acid 0.1% (v/v) in ACN (eluent B). The flow rate was set to 0.25 mL min^−1^ and elution was performed using the following gradient: 0 min/20% B, 1 min/20% B, 7 min/90% B, 9 min/90% B, 9.5 min/20% B, and 13.9 min/20% B. The injection volume was set to 1 µL and the temperature of the sample tray was maintained at 6 °C.

Positive ionisation was performed using a Heated Electro Spray Ionization (HESI) source. The spray voltage was set to 3.5 kV and nitrogen was used as a sweep, auxiliary and sheath gas. The auxiliary gas heater and the capillary temperature were set to 350 °C and 300 °C, respectively. The mass spectra were acquired in a full-mass scan as well as a targeted single ion monitoring (t-SIM). The mass spectrometer scan range was set to *m/z* 100–500 and a mass resolution (*m/Δm*) of 35,000 for the full-mass scan and 70,000 for the t-SIM, was applied. The intensities of the product ions at 326.248 *m/z*, 284.201 *m/z* and 342.243 m/z corresponding to protonated tolterodine (C_22_H_32_NO^+^), N-dealkylated tolterodine (C_19_H_26_NO^+^), and 5-hydroxymethyl tolterodine (C_22_H_32_NO_2_^+^), respectively, were used for either qualitative or quantitative analysis. Data processing was performed using QuanBrowser (Thermo Xcalibur 4.2.47, Thermo Fisher Scientific).

### Quantification of tolterodine’s volatile metabolite, acetone

#### Preparation of acetone test gas

For the calibration of acetone, a gas standard was prepared using the total evaporation method. In brief, a gas bulb (Supelco Analytical, PA, USA) with a volume of 1 L was evacuated at 60 °C in an oven (Memmert, Schwabach, Germany) for 30 min using a vacuum pump (Vacuumbrand GmbH + Co KG, Wertheim, Germany). Then 0.5 µL of acetone were injected through a septum (Thermogreen, Merck KGaA, Darmstadt, Germany) into the gas bulb by means of a 5 µL syringe (Hamilton Company, Reno, NV, USA). The pressure within the gas bulb was subsequently equalised to ambient pressure using a nitrogen-filled (99.999% purity) 3 L Teflon bag (Tedlar®, SKC Ltd., Dorset, UK). Calibration standards of acetone (between 0 nM and 6.8 nM) were prepared by diluting the gas standard to 200 mL volume with nitrogen in glass syringes (250 mL, Socorex Isba SA, Ecublens, Switzerland). Each concentration was measured in triplicate using a PTR-ToF-MS.

#### Sample preparation

After a given incubation time, 150 mL of the headspace above the cell culture were collected into a 250 mL glass syringe by exchanging the gas volume with clean nitrogen. For this purpose, two glass syringes were attached to the two sample ports of the flask, one filled with 150 mL of nitrogen and a second one serving for the collection of the headspace sample (see Fig. 2) when adding the nitrogen into the flask. For headspace containing higher levels of acetone the sample had to be diluted fivefold to avoid detector saturation. Both diluted and undiluted samples were analysed using a PTR-ToF-MS.

#### PTR-ToF-MS analysis

A PTR-ToF 6000 X2 (Ionicon Analytik GmbH, Innsbruck, Austria) was used in this study. The pressure, temperature and voltage of the drift tube were set to 2.6 mbar, 80 °C and 444 V, respectively. These settings resulted in a reduced electric field strength, the ratio of the electric field (*E*) to molecular number density (*N*) in the drift tube, of 120 Townsend (1 Td = 1 × 10^−17^ V cm^2^). The reagent ions, predominantly H_3_O^+^, were created in the hollow cathode ion source, which operated at a current of 3 mA. The sample inlet flow into the PTR-ToF-MS was set to 53 mL min^−1^. Data were acquired for at least 70 s with 1 spectrum/second.

Data obtained from the measurements were analysed using the PTR-ToF-MS Viewer software (Version 3.4.4.22, Ionicon Analytik GmbH). First, mass spectral peaks were fitted by a pseudo-Voigt profile. Subsequently, a two-point mass calibration was performed using the primary reaction ion H_3_^18^O^+^ at *m/z* 21.023 and the internal diiodebenzene standard C_6_H_5_I^+^ at *m/z* 203.943. The acetone product ion signal C_3_H_7_O^+^ at *m/z* 59.050 was normalised to a reagent ion signal ((H_3_O^+^ and (H_2_O)H_3_O^+^) of 10^6^ ions per second. Owing to detector saturation, the individual H_3_O^+^ (*m/z* 19.018) and (H_2_O)H_3_O^+^ (*m/z* 37.029) intensities were determined using the signals for H_3_^18^O^+^ at *m/z* 21.023 and the water cluster (H_2_O)H_3_^18^O^+^ at *m/z* 39.033, respectively. The average signal intensity of the normalised ion intensities was determined using 30 mass spectra for which a stable ion signal was recorded.

### Statistical analysis

All results are presented as a mean ± one standard deviation determined from at least two individual experiments in which duplicate or triplicate measurements were performed in separate wells or cell culture flasks. A one-way ANOVA with Tukey’s Multiple Comparison Test and a t-test were performed to compare the groups in which results were considered statistically significant, taken to be *p* < 0.05, using the OriginPro 2022b software (OriginLab©, Northampton, MA, USA). GraphPad Prism 5.0 (GraphPad Software Inc., San Diego, CA, USA) was used for the statistical and non-linear regression analyses to determine TC_50_ (half maximal toxic concentration) values.

Limits of detection (LOD) and limits of quantification (LOQ) were determined using calibration curves ranging from 0 µM to 0.03 µM for the LC-MS measurements and 0 nM to 1.1 nM for the PTR-ToF-MS measurements. The determination of LOD and LOQ values followed the guidelines outlined in the German industry norm DIN32645: 2008-11^41^.

## Results and discussion

### DrugBank search

Our search of DrugBank yielded 332 drugs identified as substrates of CYP3A4. Among these, 80 substrates were found to contain an isopropylamine substructure. Out of these 80 substrates, we eliminated structures for which the ketone metabolite is still connected to the substrates, such as dolasetron, and for which the ketone metabolite contains more than 8 carbon atoms, such selegiline. Thus, after the knowledge-based reduction of the search results, tolterodine was selected as a possible precursor that could be metabolised by CYP3A4 to a volatile metabolite as shown in Fig. 1.

### Determining tolterodine conversion in different CYP enzyme overexpressing HepG2 cell clones

**Fig. 3:**
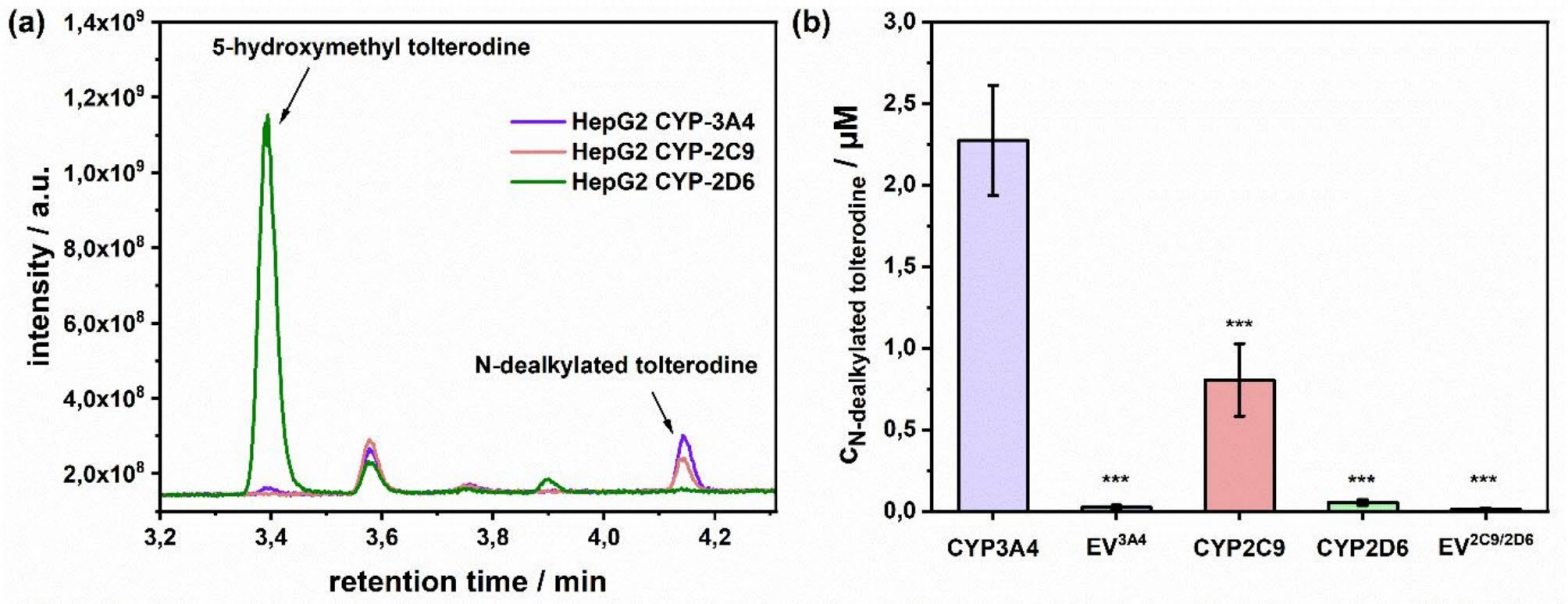
(**a**) A chromatogram illustrating the production of two major metabolites 5-hydroxymethyl tolterodine (predominantly from the CYP2D6 isoform) and N-dealkylated tolterodine (predominantly from CYP2C9 and CYP3A4). (**b**) Biotransformation of tolterodine after 4 h incubation time with three different CYP overexpressing HepG2 cell clones and the corresponding empty vector (EV) cell clones to N-dealkylated tolterodine measured by LC-MS. ***p < 0.001 in comparison to the HepG2-CYP3A4 cells.

To check the substrate’s CYP specificity, tolterodine was initially investigated using HepG2 cell clones separately overexpressing the enzymes CYP3A4, CYP2C9 and CYP2D6 in individual measurements. Figure 3a shows that N-dealkylated tolterodine at a retention time of 4.14 min is predominantly released from the CYP3A4 and CYP2C9 overexpressing cells, whereas 5-hydroxymethyl tolterodine at a retention time of 3.40 min mainly comes from the HepG2-CYP2D6 overexpressing cells (see Supplementary Fig. S1).

Concerning the quantity of N-dealkylated tolterodine, illustrated in Fig. 3b, HepG2 cells overexpressing CYP3A4 yielded the highest amount at 2.3 ± 0.3 µM. In comparison, 0.8 ± 0.2 µM N-dealkylated tolterodine resulted from the HepG2-CYP2C9 cells and even less, only 0.05 ± 0.02 µM, from the HepG2-CYP2D6 cells. The corresponding EV cell lines produced 0.02 ± 0.01 µM and 0.01 ± 0.01 µM for the CYP3A4 and CYP2C9/2D6 isoforms, respectively. Those values associated with the EV cells are to experimental error within or only slightly above the LOQ of 0.007 µM and the LOD of 0.002 µM of N-dealkylated tolterodine measured by LC-MS, and hence are negligible. These results are consistent with existing literature describing the N-dealkylated metabolite as being predominantly formed by CYP3A4 and CYP2C9 enzymes and the 5-hydroxymethyl tolterodine by CYP2D6^28^. Since the volatile metabolite, acetone can only be formed by the N-dealkylation of tolterodine to N-dealkylated tolterodine, all subsequent experiments only involved the HepG2-CYP3A4 overexpressing cells (see Fig. 1).

### Concentration-dependent effects of tolterodine on HepG2-CYP3A4 cells

**Fig. 4:**
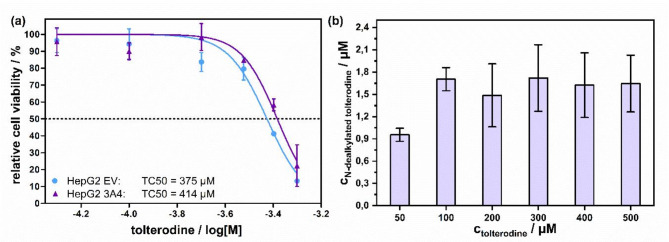
(**a**) Relative cell viability of HepG2-CYP3A4 and HepG2-EV cells after exposure to different tolterodine concentrations (up to 500 µM) was evaluated by a trypan blue exclusion assay. (**b**) Production of N-dealkylated tolterodine after treatment of HepG2-CYP3A4 cells with different tolterodine concentrations. Cell supernatants were collected after 4 h treatment and measured by LC-MS.

With respect to tolterodine being a proposed substrate for determining CYP3A4 activity via the production of its volatile metabolite, acetone, it is first necessary to test the biological effect of tolterodine exposure on cell viability. Figure 4a provides the relative cell viability after exposure to different tolterodine concentrations for HepG2-CYP3A4 overexpressing cells and their respective EV control cells. As expected, with an increasing tolterodine concentration a decrease in the proportion of living cells in both cell lines is observed. There was still a high cell viability up to 200 µM of tolterodine, whereas above 200 µM the number of living cells decreased gradually. The resulting TC_50_-values, at which 50% of the cells were dead, is determined to be 414 µM for the HepG2-CYP3A4 cells and 375 µM for the EV reference. As the values were similar in both cell lines, it can be concluded that bioconversion of tolterodine via CYP3A4 does not lead to the formation of a toxic metabolite or to the detoxification of tolterodine.

An important aim of these experiments was to test if CYP3A4-mediated metabolism of tolterodine showed also a concentration-dependent pattern. Figure 4b shows that the amount of N-dealkylated tolterodine formed in HepG2-CYP3A4 cells increased in going from 50 µM to 100 µM tolterodine, and then remained relatively constant with increasing concentrations. However, as shown in Fig. 4a, from 200 µM on, tolterodine became toxic to the cells, and thus a gradually decreasing cell number led to those similarly high levels of the tolterodine metabolite. These measurements show that in order to achieve a sufficiently high substrate conversion, while maintaining high cell viability, 100 µM tolterodine can be used as a suitable substrate concentration for our further experiments.

### Time dependent biotransformation of tolterodine in HepG2-CYP3A4 cells

**Fig. 5:**
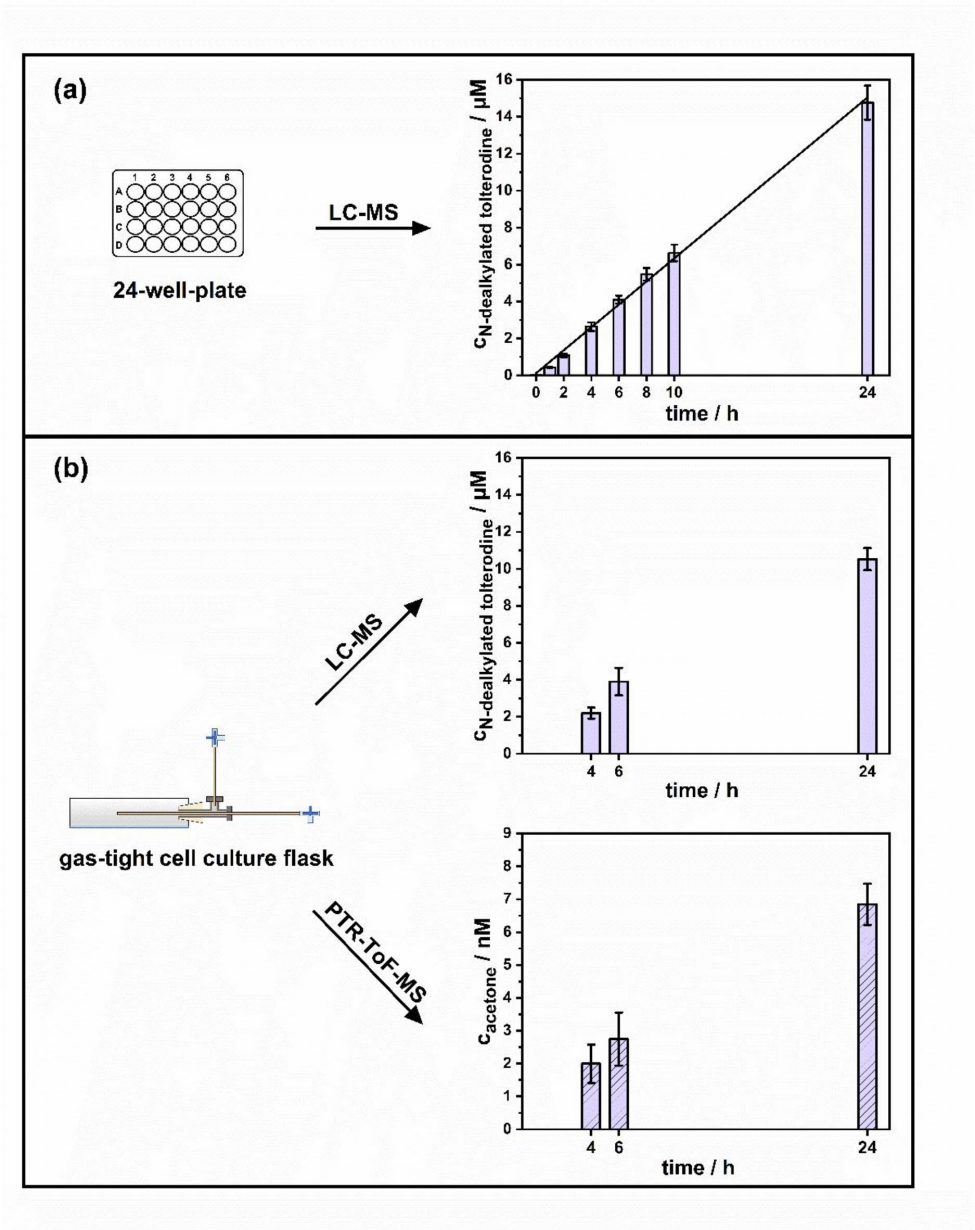
Time-dependent production of N-dealkylated tolterodine and acetone by HepG2-CYP3A4 overexpressing cells after treatment with 100 µM tolterodine (**a**) over a period of 24 h in a 24-well-plate with a linear fit (R^2^ = 0.9965) and (**b**) at 3 set time points in a cell culture flask. Cell supernatants and headspace were collected at given time points and measured by LC-MS and PTR-ToF-MS, respectively.

After treatment of HepG2-CYP3A4 cells with 100 µM of tolterodine (defined as time 0 min), the concentration of the N-dealkylated tolterodine was found to increase linearly over 24 h, as shown in Fig. 5a. This resulted in a final non-volatile metabolite concentration of 14.8 ± 0.9 µM after 24 h of incubation. In contrast, the biotransformation of tolterodine by the EV control cells produced only a negligible amount of N-dealkylated tolterodine. After 24 h of incubation, 0.04 ± 0.01 µM N-dealkylated tolterodine was formed, which is within the LOQ of 0.03 µM. This provides evidence that the biotransformation of tolterodine was mediated by the expressed CYP3A4 enzymes and was not initiated by other cellular or technical issues occurring during sample preparation or measurements.

Measurements for the biotransformation of 100 µM tolterodine to N-dealkylated tolterodine and acetone in cell culture flasks sealed with the sampling device after 4 h, 6 h and 24 h is presented in Fig. 5b. The results of the N-dealkylated metabolite agree well with those in the 24-well-plate experiment. After 4 h and 6 h, 2.2 ± 0.3 µM and 3.9 ± 0.7 µM of N-dealkylated was formed, respectively. After 24 h of biotransformation, 10.5 ± 0.6 µM of the non-volatile metabolite was measured, which is slightly lower than that found in the 24-well plate experiment. The observed differences in the liquid phase may be due to the varying aqueous volumes above the cells and the disparate cultivation conditions. The airtight sealing and lower oxygen levels may have resulted in a reduction in cell metabolism and CYP activity. The production of acetone after 4 h, 6 h and 24 h bioconversion were 2.0 ± 0.6 nM, 2.8 ± 0.8 nM and 6.9 ± 0.6 nM, respectively. The results for the EV control after 6 h and 24 h were 0.13 ± 0.05 nM and 0.20 ± 0.17 nM, respectively, which are below the LOQ of 0.26 nM but above the LOD of 0.07 nM. After 4 h, 0.26 ± 0.16 nM acetone was produced, close to the LOQ.

By comparing the amount of acetone formed to that of N-dealkylated tolterodine, it can be observed that qualitatively the trend of the respective metabolite production is consistent. However, quantitatively there is a major discrepancy by about a factor of approximately 1000. To get into the headspace, acetone has to first pass through the solution and reach an equilibrium, which is associated to the water/air partition coefficient (approximately 360 at 37 °C)^42^. The resulting differences observed could also be associated with the buffer solution used, for which its salt content and pH could influence the headspace volatile metabolite concentrations. To determine the ratio between the acetone concentrations in the buffer solution and in the headspace at equilibrium, the procedure outlined in “Equilibrium between gas and liquid phase” was performed. The selected acetone concentrations were based on the bioconversion of tolterodine, which should produce both volatile and non-volatile metabolites in a stoichiometric ratio of 1:1 (2.2 µM and 3.9 µM acetone equal to the amount of N-dealkylated metabolite formed during bioconversion after 4 h and 6 h, respectively). The acetone concentrations in the gas phase following equilibrium were measured to be 1.3 ± 0.3 nM and 2.5 ± 0.2 nM. These values are within the range of acetone levels produced in the cell culture headspace (see Fig. 5b). These results demonstrate that a noticeable difference in acetone headspace levels persisted, despite the fact that over 99% of the acetone remained in the supernatant. Nevertheless, Fig. 5b shows that the differences between the volatile and non-volatile metabolite became more pronounced after 24 h of incubation. A possible explanation for this is that for a long incubation time diffusion of acetone through the sampling port has occurred and is hence lost from the system. Independent of the lower acetone concentrations that are in the headspace, we have demonstrated that the production of both metabolites follows the same trend over time in the PTR-ToF-MS (acetone) and LC-MS (N-dealkylated tolterodine) measurements.

### 1-ABT- and ketoconazole-mediated inhibition of tolterodine bioconversion in HepG2-CYP3A overexpressing cells

**Fig. 6:**
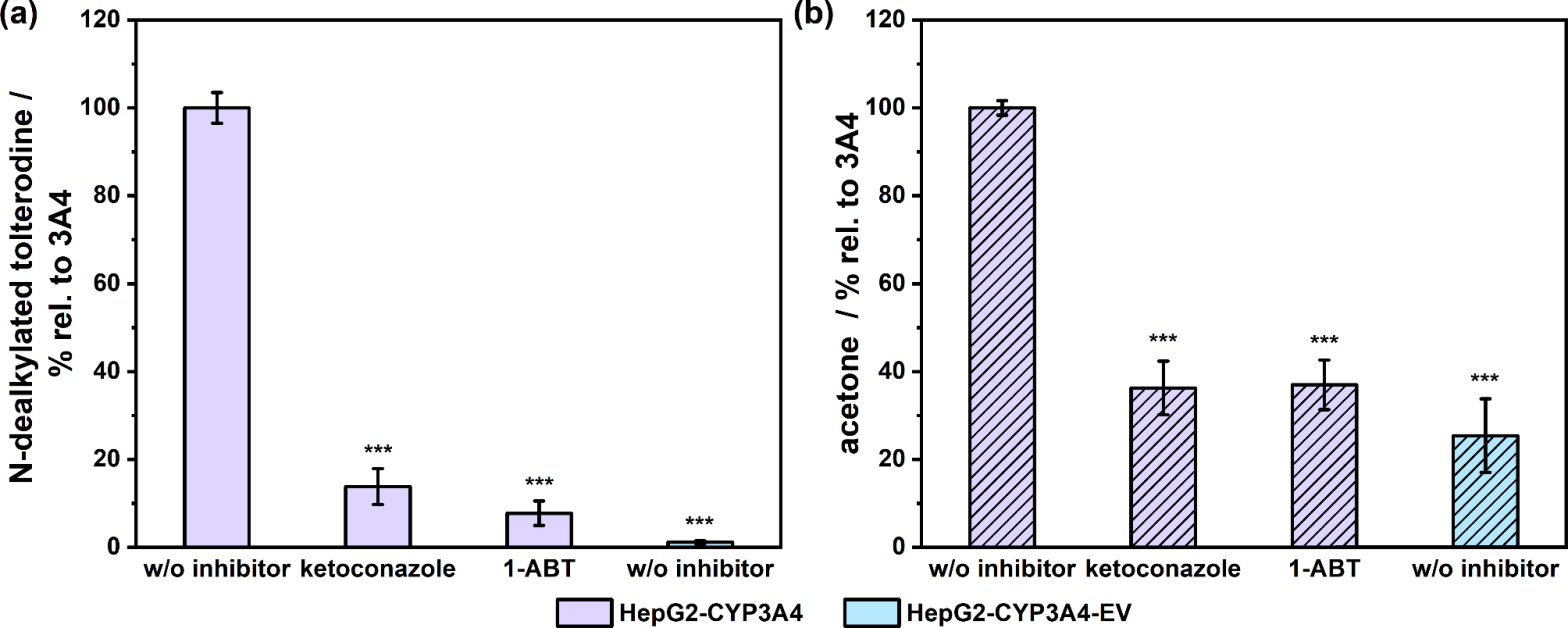
The effect of 1 mM 1-ABT and 5 µM ketoconazole on tolterodine bioconversion in HepG2-CYP3A4 cells and empty vector (EV) reference cells regarding production of (**a**) N-dealkylated tolterodine and (**b**) acetone after treatment with 100 µM tolterodine for 4 h. Cell supernatants and headspace were collected and measured by LC-MS and PTR-ToF-MS respectively. ***p < 0.001 when compared to HepG2-CYP3A4 cells treated without inhibitor.

1-ABT as a general CYP inhibitor and ketoconazole as a specific CYP3A4 inhibitor were used to prove that inhibition of CYP3A4 enzyme activity would lead to a reduced production of tolterodine metabolites. As can be seen in Fig. 6, the biotransformation to both the non-volatile and the volatile tolterodine metabolites was significantly inhibited and resulted in levels near to those of EV control cell levels. Figure 6a shows that only 7.8 ± 2.8% and 13.8 ± 4.1% of the non-volatile metabolite relative to the uninhibited reference was produced at 1 mM 1-ABT and 5 µM ketoconazole treatment, respectively. The EV reference cells showed a production of 1.2 ± 0.3% of N-dealkylated tolterodine compared to the CYP3A4 overexpressing HepG2 cells. In comparison, Fig. 6b shows that only 37.0 ± 5.6% and 36.3 ± 6.1% of acetone was formed with 1 mM 1-ABT and 5 µM ketoconazole treatment, respectively, compared to the uninhibited HepG2-CYP3A4 cells, and that the EV reference produced 25.4 ± 8.4% acetone. The difference in the inhibition observed between the headspace and liquid measurements is probably due to inaccuracies in the headspace measurements, because the liquid phase measurements demonstrated generally considerably less variation than the headspace analyses. It must be noted, however, that in both cases, CYP inhibition results in metabolite formation almost as low as shown in the EV cells, which can be regarded as a negative control for CYP3A4 activity in HepG2 cells.

It is noticeable that tolterodine bioconversion in HepG2-CYP3A4 cells was more inhibited by 1-ABT for the N-dealkylated metabolite than by ketoconazole at the concentrations tested (Fig. 6a). This is to be expected, because 1-ABT exerts inhibitory effects on all CYP enzymes, whereas ketoconazole selectively targets the 3A4 isoform. In HepG2 cells, in addition to the overexpressed CYP3A4 enzymes, a spectrum of other CYP enzymes is also expressed at low levels^11,31,32^ and, as shown in our comparative experiment between cell lines, the N-dealkylated metabolite is formed not only by CYP3A4, but also by the CYP2C9 isoform.

Several studies have confirmed that CYP3A4 is primarily responsible for the production of the N-dealkylated metabolite of tolterodine. Postlind et al.^28^ investigated the correlation between CYP activity and metabolite formation in microsomes from 10 different human liver samples. Their results demonstrated that the formation of the N-dealkylated metabolite correlated most strongly with CYP3A4 activity. Additionally, CYP2C9 and CYP2C19 were found to contribute to the formation of this metabolite, which is consistent with our findings. Moreover, another study^43^ has shown that N-dealkylation plays a significant role in the elimination of tolterodine as soon as the primary metabolic pathway, mediated by CYP2D6 and forming 5-hydroxymethyl tolterodine, is inhibited. Lastly, the influence of CYP3A4 inhibition on tolterodine pharmacokinetics has been documented. In individuals with CYP2D6 deficiency, inhibition of CYP3A4 using ketoconazole resulted in a 60% reduction in oral clearance of tolterodine and a 50% increase in its terminal half-life^44^.

## Summary and conclusions

Our comparative experiments with different CYP overexpressing HepG2 cell lines, namely CYP3A4, CYP2D6 and CYP2C9, revealed different metabolic profiles for tolterodine biotransformation. The predominant non-volatile metabolite formed by CYP2D6 enzymes is the 5-hydroxymethyl tolterodine, which is hardly formed by CYP3A4 and CYP2C9. In comparison, the CYP3A4 overexpressing cell line exhibited the highest ability to produce N-dealkylated tolterodine, which emphasised the importance of using the CYP3A4-isoform for the subsequent experiments presented in this paper.

An investigation on tolterodine impact on HepG2-CYP3A4 cell viability showed a concentration dependent decrease in cell survival, and metabolite production stagnated at concentrations higher than 100 µM tolterodine. Selection of this substrate concentration for further experiments allowed efficient tolterodine bioconversion with high cell viability. Time-dependent biotransformation of tolterodine showed a continuous increase in the production of acetone and N-dealkylated tolterodine concentrations over 24 h. In comparison, negligible metabolite production was observed in control (EV) cells, confirming the specificity of CYP3A4 enzymes in tolterodine biotransformation. Inhibition studies with 1-ABT and ketoconazole demonstrated a considerable reduction in both acetone and N-dealkylated tolterodine formation.

Concerning the relevance of acetone as a biomarker for drug response, it is of course one of the most abundant volatile organic compounds found in exhaled human breath (~ 300–1000 ppbv)^45,46^. However, a comparison of its concentration with that of the currently most used biomarker for CYP activity^13^, CO_2_, reveals a value that is approximately 1000- fold lower. Consequently, the amount of tolterodine ingested in a potential breath test can be much lower than the precursor dose required for a ^13^C-breath test, but still result in a significant rise in breath acetone levels found in an individual’s breath. A reduction in the quantity of a precursor substrate has the potential to minimise health complications and reduce the cost of clinical tests. Nevertheless, our research has not yet reached the stage of clinical application. In a clinical context it would be necessary to elaborate whether the test works when several drugs are taken at the same time, or whether the dosage needs to be adjusted.

We comment that whilst the focus of this paper has been on the production of a volatile metabolite for developing non-invasive metabolic breath tests, it would also be of diagnostic interest to detect the more specific N-dealkylated metabolite in serum as a future CYP3A4 phenotype assay.

Of importance, whilst this work confirmed that HepG2-CYP3A4 overexpressing cells are a valuable in vitro model to study CYP3A4 dependent effects, HepG2 cells lack physiological features that would more reliably reflect metabolism within the human liver. Therefore, the next step for us is to use more physiological liver models, such as primary or primary-like human hepatocytes. Furthermore, a major direction of our research is to identify and investigate substrates that yield unique volatile metabolites distinct from endogenous volatiles commonly found in exhaled breath at high levels, such as acetone.

Despite the limitations associated with this current work, this study lays the foundations for further CYP activity studies, which in turn will aid in the development of novel non-invasive assays for predicting individual drug responses, helping to pave the way for personalised medicine.

## Electronic supplementary material

Below is the link to the electronic supplementary material.

Supplementary Material 1

## Data Availability

The datasets generated during and/or analysed during the current study are available from the corresponding author on reasonable request.
