# Supplementary material for: Tolterodine is a novel candidate for assessing CYP3A4 activity through metabolic volatiles to predict drug responses
Source: Sci Rep. 2025 Jan 20;15:2462. doi: 10.1038/s41598-025-86450-9 (PMC11743777; doi:10.1038/s41598-025-86450-9)
Supplement: Supplementary file 1 — Supplementary Material 1 [file 41598_2025_86450_MOESM1_ESM.docx]

**Supplementary Material**

**Figure S 1**: Complete chromatogram of the tolterodine conversion in different CYP enzyme overexpressing HepG2 cell clones.
